# The effects of health-related food taxes on the environmental impact of consumer food purchases: secondary analysis of data from a randomised controlled trial in a virtual supermarket

**DOI:** 10.1017/S1368980024000090

**Published:** 2024-01-15

**Authors:** Michelle Eykelenboom, Derek Mersch, Alessandra C Grasso, Reina E Vellinga, Elisabeth HM Temme, Ingrid HM Steenhuis, Margreet R Olthof

**Affiliations:** 1 Department of Health Sciences, Faculty of Science, Vrije Universiteit Amsterdam, and Amsterdam Public Health Research Institute, De Boelelaan 1085, 1081 HV Amsterdam, The Netherlands; 2 Centre for Prevention, Lifestyle and Health, National Institute for Public Health and the Environment (RIVM), Bilthoven, The Netherlands

**Keywords:** Environmental impact, Food policy, Nutri-Score, Sugar-sweetened beverage tax, Sustainability

## Abstract

**Objective:**

To measure the effects of health-related food taxes on the environmental impact of consumer food purchases in a virtual supermarket.

**Design:**

This is a secondary analysis of data from a randomised controlled trial in which participants were randomly assigned to a control condition with regular food prices (*n* 152), an experimental condition with a sugar-sweetened beverage (SSB) tax (*n* 131) or an experimental condition with a nutrient profiling tax based on Nutri-Score (*n* 112). Participants were instructed to undertake their typical weekly grocery shopping for their households. Primary outcome measures were three environmental impact indicators: greenhouse gas (GHG) emissions, land use and blue water use per household per week. Data were analysed using linear regression analyses.

**Setting:**

Three-dimensional virtual supermarket.

**Participants:**

Dutch adults (≥ 18 years) who were responsible for grocery shopping in their household (*n* 395).

**Results:**

GHG emissions (–7·6 kg CO_2_-eq; 95 % CI –12·7, –2·5) and land use (–3·9 m^2^/year; 95 % CI –7·7, –0·2) were lower for the food purchases of participants in the nutrient profiling tax condition than for those in the control condition. Blue water use was not affected by the nutrient profiling tax. Moreover, the SSB tax had no significant effect on any of the environmental impact indicators.

**Conclusions:**

A nutrient profiling tax based on Nutri-Score reduced the environmental impact of consumer food purchases. An SSB tax did not affect the environmental impact in this study.

Food choices are an important determinant of both human health and environmental sustainability^(1)^. Global transition to diets high in energy-dense and ultra-processed foods, added sugar, saturated fat and red and processed meat has contributed to an increase in overweight and associated non-communicable diseases such as type 2 diabetes, CVD, musculoskeletal disorders and several types of cancer^(2)^. At the same time, food production is responsible for 26 % of total anthropogenic greenhouse gas (GHG) emissions worldwide^(3)^. Croplands and pastures together occupy more than one-third of the Earth’s land surface^(4)^. Moreover, agriculture consumes the largest amount of water of all human activities and accounts for 70 % of global freshwater withdrawals^(5)^. These food-related impacts contribute to great environmental challenges for humanity including climate change, biodiversity loss and freshwater scarcity, and it is widely acknowledged that action is needed^(6)^.

Various policies have been developed to promote healthy food choices^(7)^. There are, however, few policies combining public health and environmental sustainability objectives^(7)^. In recent years, fiscal policies received considerable attention in obesity prevention. In 2016, the WHO recommended governments to implement a tax on sugar-sweetened beverages (SSB) and to consider taxes on other unhealthy foods, for example, those high in added sugar or saturated fat^(8)^. The rationale for health-related food taxes includes substantial evidence that price is an important determinant of food choice^(8–10)^. At present, an SSB tax has been implemented in over forty countries worldwide^(11)^. Although taxes targeting a wider range of unhealthy foods and beverages are scarce^(11)^, research suggests that such taxes may have more beneficial effects on healthy food choices than taxation of SSB alone^(12–14)^.

As food choices are an important determinant of both human health and environmental sustainability^(1)^, taxing certain foods from a public health perspective may have implications for the environmental impact of food purchases as well. Clark *et al.* demonstrated that foods associated with the largest negative environmental impacts, particularly red and processed meat, are consistently associated with the largest increases in disease risk^(15)^. This implies that dietary changes towards healthier foods would generally improve environmental sustainability. However, this is not the case for all foods. For example, SSB have relatively low negative environmental impacts per serving consumed, while the impact on disease risk is relatively high^(15)^. An SSB tax implemented from a public health perspective may cause lower environmental impact if SSB are replaced by tap water, whereas replacement by milk may increase environmental impact^(16,17)^.

To the best of our knowledge, no research has been conducted to investigate the effects of an SSB tax and a nutrient profiling tax on the environmental impact of consumer food purchases. Addressing the environmental impact of public health policies is important to prevent the undermining of environmental sustainability^(18,19)^. In addition, if health-related food taxes are associated with lower environmental impacts, the environmental sustainability argument could be used in the framing of health-related food taxes to improve their adoption^(20)^. Therefore, the aim of this study is to measure the effects of an SSB tax and a nutrient profiling tax on the environmental impact of consumer food purchases in a Dutch virtual supermarket setting. A previous analysis of the data showed that both taxes decreased SSB purchases and that the nutrient profiling tax also increased the overall healthiness and decreased the energy content of consumer food purchases^(14)^.

## Methods

The present study conducted secondary data analyses on data from a randomised controlled trial designed to investigate the effects of an SSB tax and a nutrient profiling tax on SSB and healthy food purchases in a Dutch virtual supermarket setting^(14)^. More detailed information on the study design, virtual supermarket, participants and recruitment and procedures can be found elsewhere^(14)^.

### Study design

Participants were randomly assigned to one of the following conditions: (i) a control condition with regular food prices, (ii) an experimental condition with an SSB tax or (iii) an experimental condition with a nutrient profiling tax. In the SSB tax condition, SSB were taxed on a scheme similar to the UK’s Soft Drinks Industry Levy. This implies that beverages containing 5–8 g of sugar per 100 ml were taxed €0·21 per litre and beverages containing 8 g of sugar or more per 100 ml were taxed €0·28 per litre^(11)^, which corresponded to an average price increase of 22% for the beverages liable for the tax. Milk-based drinks, milk substitute drinks, alcohol substitute drinks and 100 % fruit juices without added sugar were exempted from the tax in line with the Soft Drinks Industry Levy^(11)^. A total of thirty-four SSB were taxed in the SSB tax condition (see Table 1). In the nutrient profiling tax condition, energy-dense and nutrient-poor foods and beverages were taxed using the nutrient profiling scheme Nutri-Score^(21)^. Nutri-Score is a five-point, colour-coded scale ranging from dark green (associated with the letter ‘A’) to red (associated with the letter ‘E’), with ‘A’ representing the healthiest score and ‘E’ representing the unhealthiest score^(21)^. Products with the label ‘D’ or ‘E’ were classified as unhealthy and the prices of these products were increased by 20%. A total of 225 food and beverage products were taxed in the nutrient profiling condition (see Table 1). Participants in the experimental conditions were informed about the tax before entering the virtual supermarket with a notification. The notification was tailored to the condition; ‘In the virtual supermarket, beverages high in sugar are taxed’ or ‘In the virtual supermarket, unhealthy products high in sugar, fat and/or salt (such as biscuits, sweets, snacks and soft drinks) are taxed’.

**Table 1:** Overview of food groups and the number of taxed products in the experimental conditions in the virtual supermarket

Food groups*	Total products	Taxed products in the SSB tax condition		Taxed products in the nutrient profiling tax condition	
	*n*	*n*	%	*n*	%
Animal-based foods	127	–		64	50·4
Meat and poultry	25	–		11	44·0
Cold meat cuts	19	–		18	94·7
Cheese	20	–		19	95·0
Milk and milk products	51	–		15	29·4
Eggs	2	–		–	
Fish	10	–		1	10·0
Plant-based foods	174	–		30	17·2
Potatoes and tubers	11	–		–	
Bread	26	–		4	15·4
Fruits	18	–		–	
Vegetables	42	–		1	2·4
Herbs and spices	27	–		16	59·3
Nuts and seeds	9	–		2	22·2
Meat and dairy substitutes	11	–		4	36·4
Legumes	4	–		–	
Cereals and cereal products	26	–		3	11·5
Beverages	103	34	33·0	34	33·0
Alcoholic beverages	17	–		–	
Non-alcoholic beverages	86	34	39·5	34	39·5
Miscellaneous foods	176	–		96	54·5
Miscellaneous	2	–		–	
Savoury bread spreads	9	–		4	44·4
Savoury sauces	22	–		8	36·4
Savoury snacks	22	–		13	59·1
Pastry and biscuits	31	–		22	71·0
Fats and oils	10	–		8	80·0
Mixed dishes	23	–		–	
Soups	9	–		–	
Sugar, sweets and sweet sauces	48	–		41	85·4
Total	580	34	5.9	224	38·6

SSB, sugar-sweetened beverage.

* Classification from the Dutch Food Composition Database (NEVO)^(24)^.

### Setting: the virtual supermarket

The study was conducted using a Dutch virtual supermarket^(22)^, which is a three-dimensional software application simulating the in-store environment of a real supermarket. Validation against real-life shopping data demonstrated that food purchasing behaviour in the virtual supermarket accurately represents real-life food purchasing behaviour^(23)^. The original version of the Dutch virtual supermarket of Waterlander *et al.*^(22)^ was updated in 2019^(14)^. The virtual supermarket contained 580 different food and beverage products. Food prices and product weights were obtained from the website of the leading supermarket chain in the Netherlands in February 2020. All food and beverage products were linked to data on food categories and nutritional composition derived from the online Dutch food composition database (NEVO online version 2019/6.0)^(24)^. Nutri-Scores were calculated using a calculation tool for the original algorithm of the French National Public Health Agency^(21)^.

### Participants and recruitment

Participants were eligible to participate in the study if they were 18 years or older, had a good command of the Dutch language, were largely or totally responsible for grocery shopping in their household and had access to a laptop or computer. Participants were recruited by an online panel called ‘Panel Inzicht’ between June and August 2020. Panel Inzicht is one of the largest online research panels in the Netherlands and has more than 100 000 members^(25)^. The study was conducted according to the guidelines laid down in the Declaration of Helsinki and was evaluated by the Research Ethics Review Committee of the Faculty of Sciences, Vrije Universiteit Amsterdam (reference 20205). The study protocol was registered in the Netherlands Trial Register (NTR) (registration number NL8616). All participants provided informed consent.

### Procedures

Participants were randomised by the research panel using a computer-generated list of log-in codes for the virtual supermarket. The log-in codes corresponded with allocation to either the control or one of the experimental conditions. After log-in, participants were asked about their household size and household composition to determine a household-specific weekly grocery shopping budget based on data derived from the National Institute for Family Finance Information^(26)^. Participants were instructed to undertake their typical weekly grocery shopping for their households. When finished shopping, participants moved to the cash register and were directed to a closing questionnaire.

### Measures

The environmental impact of the food and beverage products was derived from the Dutch Life Cycle Assessment (LCA) Food database^(27)^. The LCA approach takes into account all life cycle stages of a product from cradle to plate, including primary production, processing, primary packaging, distribution, retail, supermarket, storage, preparation by the consumer (e.g. cooking) and incineration of packaging waste^(28)^. The functional unit used in the LCA Food database is 1 kg of the product. Food and beverage products were linked to the LCA via NEVO codes. The weights of the products in the virtual supermarket were used to calculate the environmental impact per unit food item. The LCA Food database contains data on approximately 250 foods, which cover more than 75 % of the daily food consumption in the Netherlands^(28)^. A total of 173 products in the virtual supermarket could be directly matched with their corresponding environmental impact based on NEVO codes. The environmental impacts of the remaining 407 products were estimated by attributing LCA data from similar products. Vellinga *et al.* matched products based on similarities in food properties, production systems and ingredient composition^(29)^. Standardised recipes from the Dutch food composition database (NEVO online version 2019/6.0) were used for composite dishes^(24)^. If not available, recipes were based on label information.

Primary outcome measures were food-related GHG emissions, land use and blue water use per household per week. The indicator GHG emissions is highly correlated with other environmental impact indicators and is therefore used as a proxy^(28)^. In the Dutch LCA Food database, blue water use and land use have, however, the weakest correlation with GHG emissions^(28)^ and are therefore additionally included as environmental impact indicators in this study. GHG emissions, expressed in kilograms of carbon dioxide equivalents (kg CO_2_-eq), refer to all emissions produced throughout the life cycle of a product that contribute to global warming, including emissions of carbon dioxide, methane and nitrous oxide^(27)^. Land use, in square metres per year (m^2^/year), refers to the use and transformation of land surfaces for agriculture^(27)^. In land use calculations for animal products, land used for producing feed and roughages (e.g. grass, soybeans and maize) consumed by animals is considered. However, land use calculations do not consider land used for grazing pastures and stables. Blue water use, in cubic metres (m^3^), refers to the use of freshwater, sourced from surface or groundwater resources, for irrigation during the cultivation of crops^(27)^. Notably, blue water use does not account for water directly consumed by animals. Information on the amount of food items purchased within the food groups meat, milk and milk products, cheese, non-alcoholic beverages and fruit is also presented, as these food groups contribute most to the environmental impact of the Dutch diet^(28)^.

In the closing questionnaire, data were collected on participant characteristics such as age (years), sex (female, male), educational level (low, moderate, high), household income (low, medium, high), height (m) and weight (kg) and dietary preference (carnivore, flexitarian, vegetarian, pescatarian, vegan) (see Table 2). Educational level was classified into low (elementary, lower secondary or lower vocational), moderate (higher secondary or intermediate vocational) and high (higher vocational or university) educational level based on the standard classification from Statistics Netherlands^(30)^.

**Table 2:** Descriptive statistics of participant characteristics

	Total (*n* 395)		Control condition (*n* 152)		SSB tax condition (*n* 131)		Nutrient profiling tax condition (*n* 112)	
	*n* or mean	% or sd	*n* or mean	% or sd	*n* or mean	% or sd	*n* or mean	% or sd
Age (years), mean and sd	48·5	15·7	48·6	16·3	48·4	15·2	48·3	15·6
Sex, *n* and %								
Female	216	54·7	78	51·3	81	61·8	57	50·9
Male	179	45·3	74	48·7	50	38·2	55	49·1
Educational level, *n* and %								
Low	66	16·7	20	13·2	21	16·0	25	22·3
Moderate	144	36·5	44	28·9	57	43·5	43	38·4
High	185	46·8	88	57·9	53	40·5	44	39·3
Household monthly income (gross in €), *n* and %								
Low (0–2000)	107	27·1	38	25·0	36	27·5	33	29·5
Medium (2000–3000)	97	24·6	37	24·3	31	23·7	29	25·9
High (3000+)	191	48·4	77	50·7	64	48·9	50	44·6
BMI (kg/m^2^)*, mean and sd	26·7	5·8	27·5	6·0	26·4	5·7	26·0	5·4
Weight status*, *n* and %								
BMI < 25 kg/m^2^	179	46·3	65	43·0	62	48·8	52	47·7
Overweight	128	33·1	49	32·5	38	29·9	41	37·6
Obese	80	20·7	37	24·5	27	21·3	16	14·7
Dietary preference, *n* and %								
Carnivore	253	64·1	95	62·5	83	63·4	75	67·0
Flexitarian	119	30·1	48	31·6	42	32·1	29	25·9
Vegetarian	14	3·5	8	5·3	2	1·5	4	3·6
Pescatarian	8	2·0	1	0·7	4	3·1	3	2·7
Vegan	1	0·3	–		–		1	0·9
Household size, mean and sd	2·3	1·2	2·3	1·2	2·4	1·3	2·4	1·2
% of shopping budget spent, mean and sd	82·1	21·1	84·1	19·8	82·2	21·2	79·3	22·4

SSB, sugar-sweetened beverage.

* *n* 387.

### Statistical analyses

Descriptive statistics were used to describe the participant characteristics and the environmental impact indicators for the total sample and the three research conditions separately. The environmental impact indicators followed a normal distribution and were analysed using linear regression analyses. It was examined whether educational level or income modified the effect of the food taxes on the environmental impact indicators because research suggests that food taxes may be more effective in reducing the purchases of taxed products among lower socio-economic groups^(9)^. Effect modification was tested by including educational level or income, the research conditions and interaction terms between the research conditions and educational level or income in the unadjusted regression models. As none of the interaction terms was statistically significant (*P* > 0·05), the results were not stratified by educational level or income. Subsequently, two regression models were made for each outcome measure: model 1 was adjusted for household size as it was proven to be a strong predictor of the environmental impact indicators, and model 2 was additionally adjusted for sex, educational level and BMI to correct for imbalances in these characteristics between the research conditions. Participants who purchased less than or equal to five different products in the virtual supermarket were excluded from all analyses as this was considered not representative of a total household weekly food shopping basket. Also, participants with extreme outliers (more than 3 * IQR below Q1 or above Q3) in any of the environmental impact indicators were excluded from all analyses. Data were analysed using the software IBM SPSS Statistics version 28. All statistical tests were two-sided and were considered statistically significant at *P* < 0·05.

## Results

**Fig. 1:**
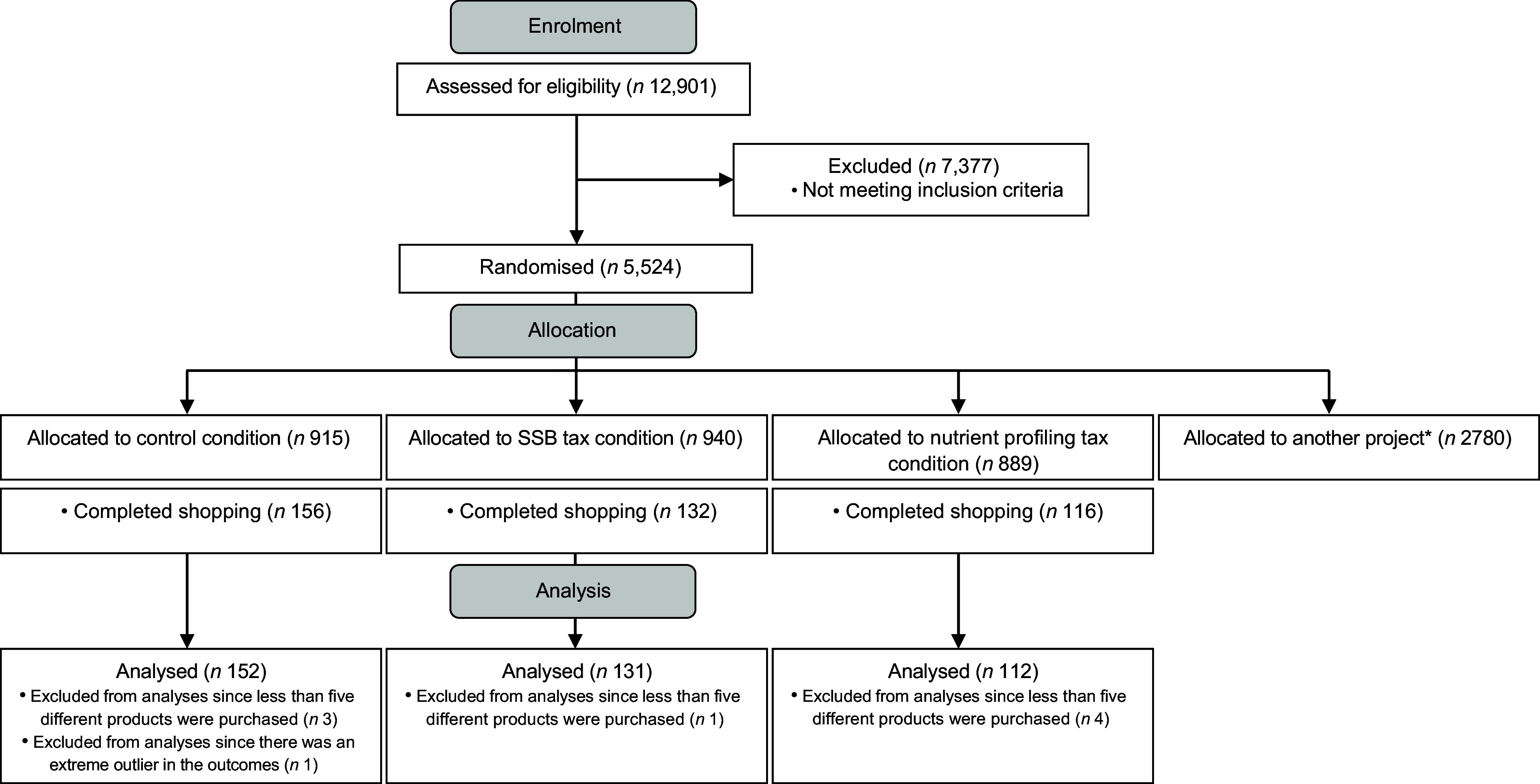
Flow chart of enrolment and allocation of the study participants for secondary analysis. * 2780 participants were randomised for the purpose of another project^(29)^

A total of 150 514 panel members were invited to participate of whom 12 901 completed the screening questionnaire and 5 524 were eligible for inclusion (Fig. 1). Of the participants, 2 744 were randomly assigned to one of the research conditions of this study. A total of 395 participants were included in the analyses. Participant characteristics are shown in Table 2. The mean age of the participants was 48·5 years (sd 15·7), 54·7 % were female and 46·8 % had a high level of education. The average household size was 2·3 persons (sd 1·2).

GHG emissions of the food purchases were on average 61·6 kg CO_2_-eq (sd 27·9) per household per week in the control condition, 60·5 kg CO_2_-eq (sd 30·0) in the SSB tax condition and 55·5 kg CO_2_-eq (sd 26·1) in the nutrient profiling tax condition (Table 3). In the fully adjusted model, GHG emissions were on average 7·6 kg CO_2_-eq (95 % CI –12·7, –2·5) per household per week lower for the food purchases of participants in the nutrient profiling tax condition than for those in the control condition (Table 4). The SSB tax had no statistically significant effect on food-related GHG emissions.

**Table 3:** Descriptive statistics of the environmental impact of the total household weekly food shopping basket and the amount of food items purchased by food group

	Total (*n* 395)		Control condition (*n* 152)		SSB tax condition (*n* 131)		Nutrient profiling tax condition (*n* 112)	
	Mean	sd	Mean	sd	Mean	sd	Mean	sd
Environmental impact								
GHG emissions (kg CO_2_-eq), mean and sd	59·5	28·1	61·6	27·9	60·5	30·0	55·5	26·1
Land use (m^2^/year), mean and sd	38·3	19·4	38·8	18·9	39·7	21·3	36·0	17·5
Blue water use (m^3^), mean and sd	1·7	1·1	1·7	1·0	1·6	1·1	1·6	1·1
Food purchases								
Total (*n*)	39·7	17·6	40·1	16·8	40·5	18·5	38·2	17·6
Meat (*n*)	4·1	2·8	4·2	2·9	4·1	2·9	3·8	2·4
Milk and milk products (*n*)	3·9	3·1	4·0	2·9	4·1	3·3	3·8	3·1
Cheese (*n*)	1·1	1·1	1·1	1·1	1·1	1·1	1·0	1·1
Non-alcoholic beverages (*n*)	3·4	2·8	3·4	2·8	3·5	2·7	3·3	2·9
Fruit (*n*)	2·1	1·7	2·1	1·6	2·1	1·7	2·0	1·9

SSB, sugar-sweetened beverage; GHG, greenhouse gas.

**Table 4:** Effects of the experimental conditions on GHG emissions, land use and blue water use of the total household weekly food shopping basket using linear regression analyses

		SSB tax condition			Nutrient profiling tax condition		
		*B* ‡	95 % CI	*P*	*B* ‡	95 % CI	*P*
GHG emissions (kg CO_2_-eq)	Model 1*	–3·4	–8·2, 1·4	0·159	–7·3	–12·4, –2·3	0·004§
	Model 2†	–4·3	–9·2, 0·6	0·083	–7·6	–12·7, –2·5	0·004§
Land use (m^2^/year)	Model 1*	–0·6	–4·1, 2·9	0·731	–3·6	–7·3, 0·0	0·050
	Model 2†	–1·3	–4·8, 2·3	0·480	–3·9	–7·7, –0·2	0·038§
Blue water use (m^3^)	Model 1*	–0·1	–0·3, 0·1	0·289	–0·1	–0·4, 0·1	0·214
	Model 2†	–0·1	–0·3, 0·1	0·286	–0·1	–0·4, 0·1	0·271

SSB, sugar-sweetened beverage; GHG, greenhouse gas.

* Adjusted for household size.

† Adjusted for household size, sex, educational level and BMI.

‡ Compared with the control condition.

§ *P* < 0.05.

Land use of the food purchases was on average 38·8 m^2^/year (sd 18·9) per household per week in the control condition, 39·7 m^2^/year (sd 21·3) in the SSB tax condition and 36·0 m^2^/year (sd 17·5) in the nutrient profiling tax condition (Table 3). In the fully adjusted model, land use was on average 3·9 m^2^/year (95 % CI –7·7, –0·2) per household per week lower for the food purchases of participants in the nutrient profiling tax condition than for those in the control condition (Table 4). The SSB tax had no statistically significant effect on food-related land use.

Blue water use of the food purchases was on average 1·7 m^3^ (sd 1·0) per household per week in the control condition, 1·6 m^3^ (sd 1·1) in the SSB tax condition and 1·6 m^3^ (sd 1·1) in the nutrient profiling tax condition (Table 3). The SSB tax and the nutrient profiling tax had no statistically significant effect on food-related blue water use (Table 4).

## Discussion

The aim of this study was to measure the effects of an SSB tax and a nutrient profiling tax on the environmental impact of consumer food purchases in a Dutch virtual supermarket setting. We demonstrated that the nutrient profiling tax reduced GHG emissions and land use of consumer food purchases. Blue water use was not affected by the nutrient profiling tax. Moreover, the SSB tax did not affect any of the environmental impact indicators of consumer food purchases in this study.

Contrary to the SSB tax condition, in the nutrient profiling tax condition a considerable amount of food products that contribute most to the environmental impact of the Dutch diet were taxed, which may explain the results of this study. The main contributors to GHG emissions of the Dutch diet are meat, dairy, cheese and non-alcoholic beverages^(28)^. The primary sources of non-alcoholic beverage-related GHG emissions vary by age group. For adults, coffee and tea are the most important contributors, while soft drinks are the most important beverages contributing to non-alcoholic beverage-related GHG emissions for children^(28)^. Within the four food groups, 66 %, 29 %, 95 % and 40 % of the products were taxed in the nutrient profiling tax condition, respectively. The purchases within these food groups were all in the expected direction – that is, lower amounts of these taxed foods were purchased compared with the control condition, although not statistically tested. The main contributors to blue water use of the Dutch diet are non-alcoholic beverages, fruits and meat^(28)^. Fruits were not taxed in the nutrient profiling tax condition. Although not statistically tested, it seems that participants did not purchase more fruits in the nutrient profiling tax condition than in the control condition, which may explain why the nutrient profiling tax did not increase blue water use. The purchases of meat and non-alcoholic beverages were reduced, but reductions were probably too small to affect blue water use. The SSB tax targeted a single food group that has a relatively low negative environmental impact per serving^(15)^. The reduction in the purchases within this single food group was probably too small to identify statistically significant effects on the environmental impact of the total food purchases.

We are not aware of other studies that have investigated the effects of an SSB tax and a nutrient profiling tax on the environmental impact of consumer food purchases. There are, however, studies that investigated the environmental impact of other food taxes. A Dutch virtual supermarket study by Vellinga *et al.* showed that (i) 30 % higher meat prices and (ii) 30 % higher meat prices combined with an information nudge about the environmental impact of meat production could reduce meat purchases^(29)^. The study further showed that food-related GHG emissions were, respectively, 56·3 and 54·4 kg CO_2_-eq per household per week in these conditions, which was lower than in the control condition (62·3 kg CO_2_-eq per household per week) (not statistically tested). Food-related GHG emissions in the higher meat price conditions were comparable to food-related GHG emissions in the nutrient profiling tax condition in our study (55·5 CO_2_-eq per household per week). A social cost–benefit analysis in the Netherlands demonstrated that 30 % higher meat prices could result in beneficial health and environmental impacts^(31)^. Overall, 30 % higher meat prices could lead to a net benefit for society between €4100 and €12 300 million over 30 years. Briggs *et al.* demonstrated that a food-based GHG emission tax has the potential to reduce GHG emissions and may have health co-benefits^(32)^. We demonstrated that a nutrient profiling tax targeting foods and beverages from a health perspective may have environmental co-benefits.

### Strengths and limitations

A strength of this study is that it is the first study that investigated the effects of an SSB tax and a nutrient profiling tax on the environmental impact of consumer food purchases. The effects were investigated in a controlled setting. A validation study showed that food shopping behaviour in the virtual supermarket is highly comparable to real-life food shopping behaviour^(23)^. Another strength is the use of the Dutch LCA Food database to estimate the environmental impact of food purchases. LCA data cover more than 75 % of the daily food consumption in the Netherlands and the environmental impact estimates as obtained by the LCA approach include all life cycle stages of a product^(28)^. Therefore, the used environmental data provide a comprehensive understanding of the environmental impact of consumer food purchases. The focus on total food purchases rather than on a single food group further contributed to a comprehensive understanding of the environmental impact of consumer food purchase behaviour, as the substitution effects of the health-related food taxes were taken into account.

This study also has limitations. First, this study conducted secondary analyses on data from a randomised controlled trial designed to investigate the effects of an SSB tax and a nutrient profiling tax on SSB and healthy food purchases^(14)^. Therefore, the study was not statistically powered on environmental impact indicators. Nevertheless, our sample was relatively large compared with other virtual supermarket studies. Second, despite efforts to minimise dropout, a significant number of participants dropped out after randomisation. Reasons for drop out, particularly among older participants and participants with a lower educational level, are unclear but may relate to lower computer literacy^(14)^. Third, we only have insight into the environmental impact of the total food purchases and not into the environmental impact of purchases within specific food groups. It was therefore not possible to assess the effects of the taxes on the environmental impact of purchases within specific food groups. Fourth, it should be considered that the environmental impact estimates as obtained by the LCA approach included all life cycle stages of a product, including the stages after food purchasing – for example, storage at home, consumer preparation and incineration of packaging waste^(28)^. Although the stages after food purchasing generally make, depending on the type of food, only a minor contribution to the overall environmental impact of food^(33)^, the LCA approach could still overestimate the environmental impact of food purchases. Finally, food products for which no primary LCA data were available had to be matched to similar foods based on similarities in food properties, production systems and ingredient composition, which comes with data uncertainty. Nevertheless, LCA data cover more than 75 % of the daily food consumption in the Netherlands^(28)^ and this method enabled us to determine the environmental impact of the total food purchases.

### Implications for practice

The common ground of human health and environmental sustainability emphasises the need to identify double-duty actions, which simultaneously act on both issues^(34)^. A previous analysis of data from our randomised controlled trial demonstrated that an SSB tax and a nutrient profiling tax could have beneficial effects on consumer food purchases from a public health perspective^(14)^. The current analysis showed that the nutrient profiling tax may also reduce the environmental impact of consumer food purchases. Combining the results of both analyses, it seems that the nutrient profiling tax may address both human health and environmental sustainability objectives and could therefore act as double-duty action. This insight could be used in the framing of a nutrient profiling tax to gain political and public attention^(20)^. As the public health argument has not always been decisive in the decision-making process, coupling health-related food taxes to an alternative societal issue such as environmental sustainability may open a window of opportunity for policy change^(20)^.

## Conclusions

In conclusion, a nutrient profiling tax targeting foods and beverages from a health perspective reduced the environmental impact of consumer food purchases. An SSB tax did not affect the environmental impact indicators in this study. Future high-quality studies are needed to confirm these findings. Such studies will contribute to a greater understanding of the environmental impact of public health policies, which is important to prevent the undermining of environmental sustainability and to inform policymakers on double-duty actions.
